# Supplementary material for: Muc5b-deficient mice develop early histological lung abnormalities
Source: Biol Open. 2019 Nov 7;8(11):bio046359. doi: 10.1242/bio.046359 (PMC6899002; doi:10.1242/bio.046359)
Supplement: Supplementary information [file biolopen-8-046359-s1.pdf]

Suppl. Figures

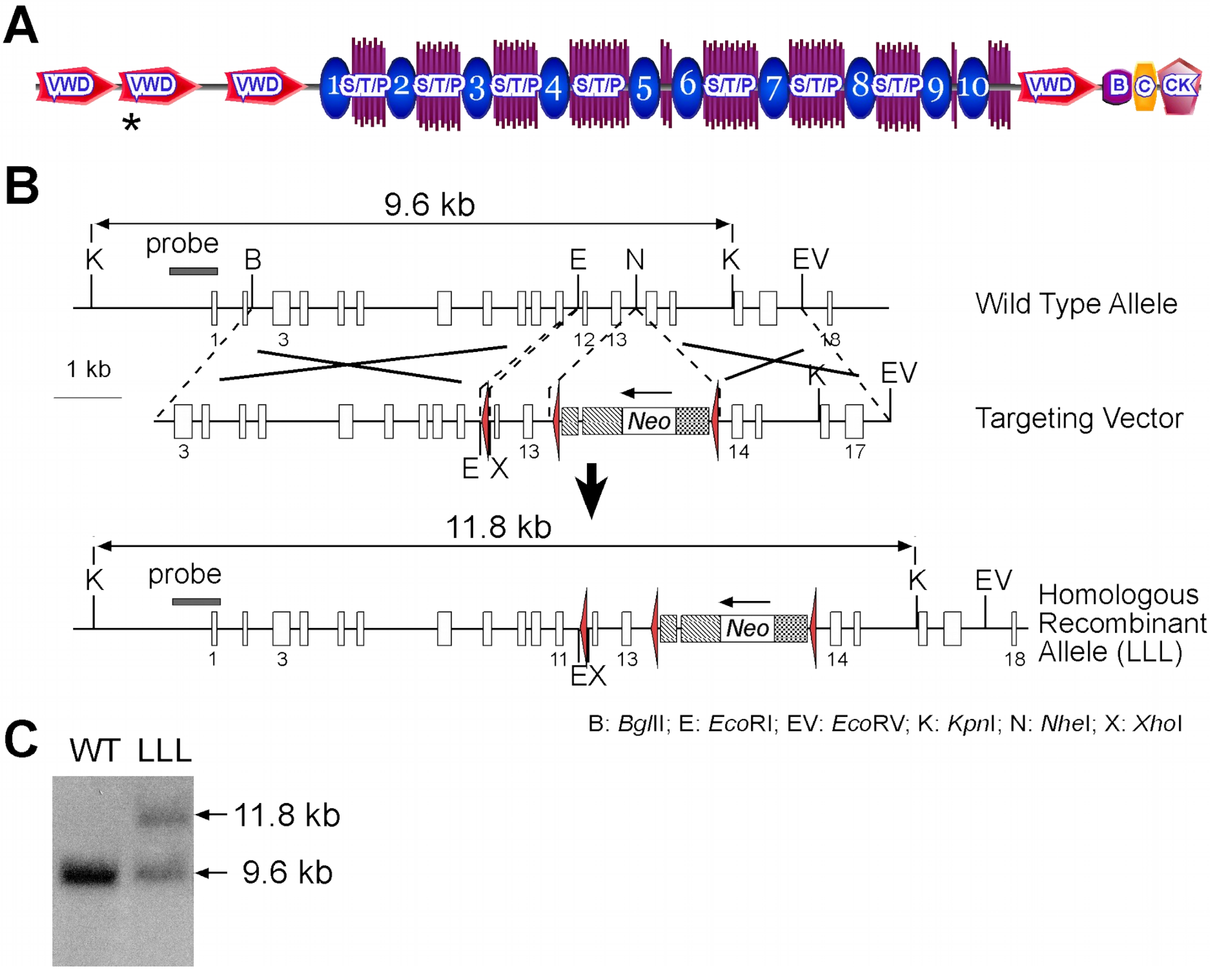

Figure S1. Targeted disruption of the *Muc5b* gene. (A) Schematic domain structure of mouse Muc5b protein. Mouse Muc5b protein is composed of amino- and carboxy-terminal regions that share similar domains with von-Willebrand factor (vW-B, -C, -CK and -D). The central part of Muc5b contains large regions enriched in Ser, Thr and Pro (S/T/P), which are extensively substituted with *O*-glycans. A highly conserved domain (oval blue) named CYS-domain is found 10 times in Muc5b and is linked to or interrupts the Ser/Thr/Pro regions. The asterisk indicates the targeted region used to invalidate *Muc5b*. **(B)** Structure and partial restriction enzyme map of the genomic segment of *Muc5b* used to generate the targeting vector. Empty boxes indicate exons and some of them are numbered. In the targeting vector, exons 12 and 13 are flanked by loxP sites and a NEO cassette flanked by a third loxP site was inserted into intron 13, in the opposite transcriptional orientation to that of *Muc5b*, as indicated by the arrow. **(C)** Southern blot analysis of DNA isolated from a control ES clone (wild-type, WT) and the positive embryonic stem cell clone (LLL). DNA was digested with *Kpn*I and hybridized with the 5’ probe (indicated by a gray box in (B)). The 9.6 and 11.8 kb bands correspond to the WT and mutant allele (LLL allele), respectively.

Biology Open • Supplementary information

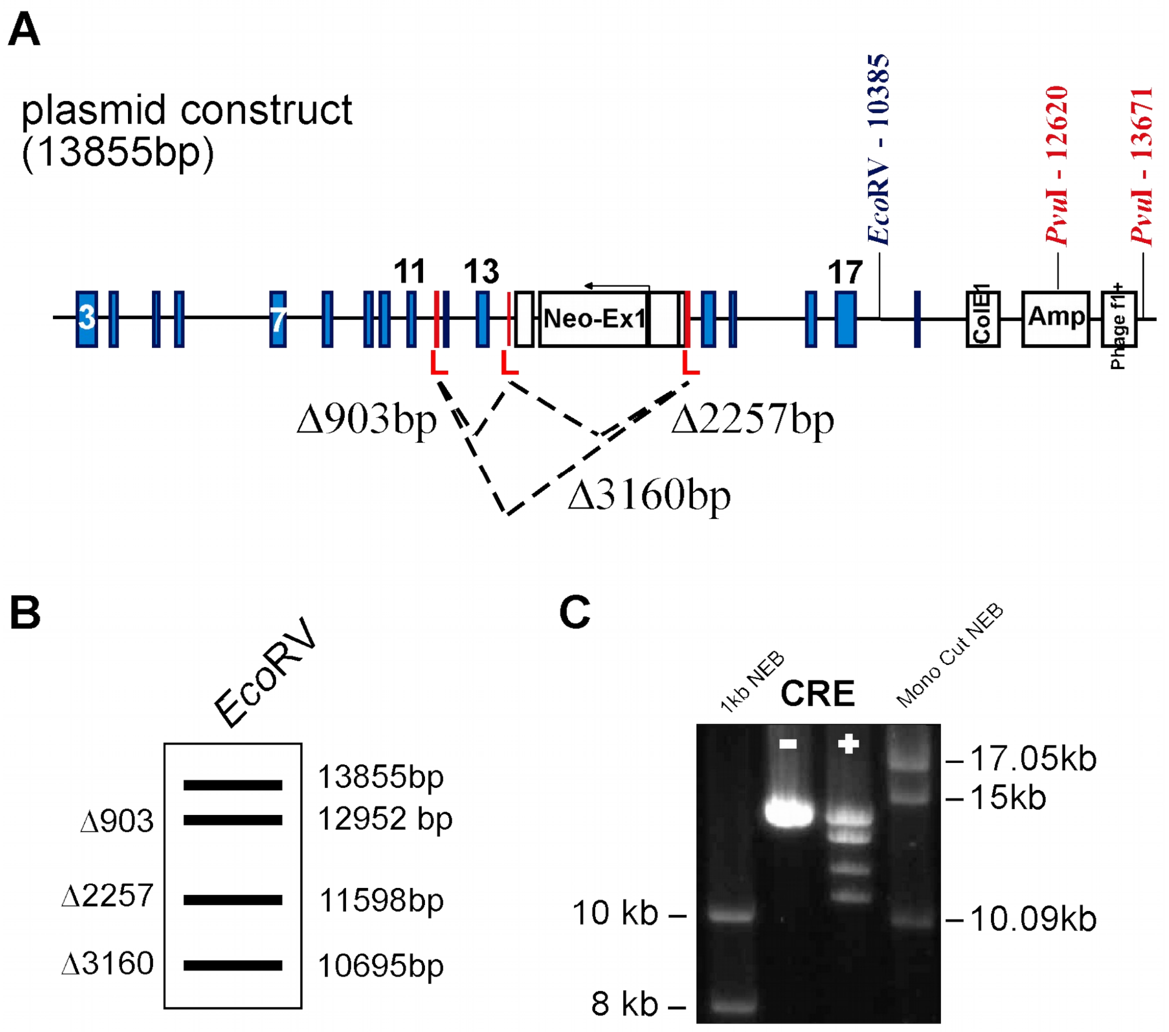

Figure S2. The three loxP sites are in the same orientation. (A) Schematic representation of the targeting vector. The three deletion (Δ) events are represented. **(B)** Predicted profile of *Eco*RV-linearised plasmid on agarose gel after Cre recombination if the three loxP sites have the same orientation. **(C)** Following incubation (+) of the circular plasmid construct with Cre recombinase (New England Biolabs) for 3 h, the DNA was digested with *Eco*RV and analyzed on a 0.6% agarose gel in comparison with the plasmid construct without Cre recombinase (–). Plasmid DNA with Cre (+) showed one band corresponding to the unrecombined DNA (13.9 kb) and three lower bands that correspond to the three possible recombination events depicted in (A). The two DNA ladders are the 1 kb-ladder and the monocut-ladder from New England Biolabs.

Biology Open • Supplementary information

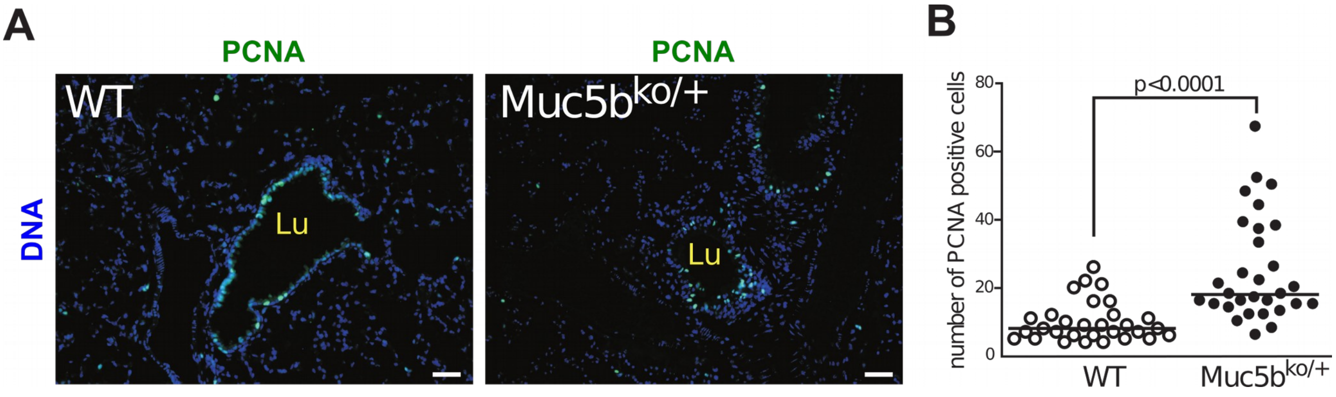

Figure S3. Increase cell proliferation in Muc5b$^{ko/+}$ mice with respiratory distress. (A) Representative immunofluorescence pictures of paraffin-embedded lung section of a wild-type (WT) and a Muc5b$^{ko/+}$ mouse stained with anti-proliferative cell nuclear antigen (PCNA) antibody. Lu = lumen; Scale bars = 50 µm. **(B)** The number of PCNA-positive cells was evaluated in the lungs of three WT (white circles; 10 bronchi per mouse) and three Muc5b$^{ko/+}$ (black circles; 10 bronchi per mouse) mice. Data were analyzed using the Wilcoxon-Mann-Whitney test.

Biology Open • Supplementary information

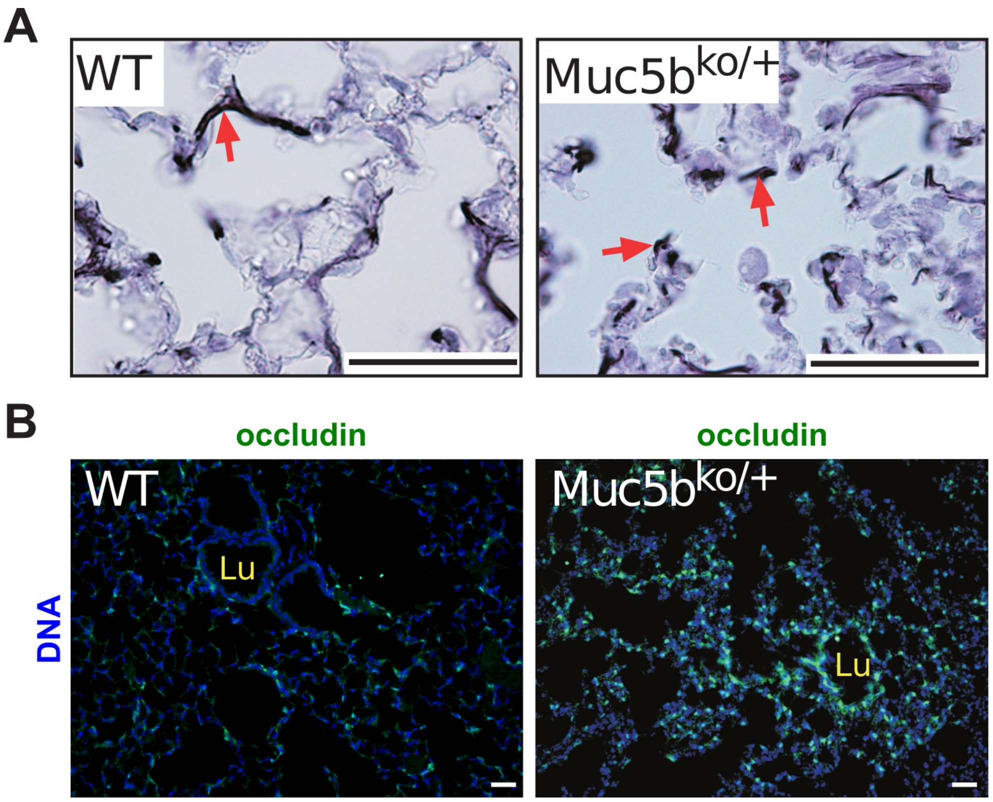

Figure S4. Disorganization of tight junctions of Muc5b$^{ko/+}$ mice with respiratory distress. (A) Orcein staining showing disorganization of elastin fibers (red arrows) in Muc5b$^{ko/+}$ mice. **(B)** Representative immunofluorescence pictures of paraffin-embedded lung sections stained with anti-occludin antibody. Lu = lumen; Scale bars = 50 µm.

Biology Open • Supplementary information

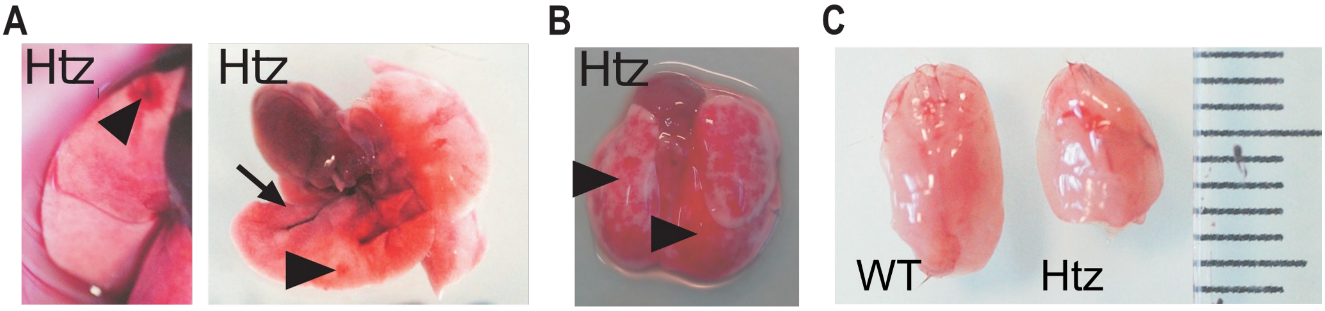

Figure S5. Heterozygous mice displayed abnormal lung morphology and atrophy of the salivary glands. (A) Macroscopic examination of a lungs from a Muc5b$^{ko/+}$ (Htz) mouse without respiratory distress. Arrowheads indicate areas of hemorrhage. **(B)** The hemorrhagic area is larger for a mouse with respiratory distress. **(C)** Macroscopic examination of salivary glands from wild-type (WT) and Htz mice. Htz mice exhibited atrophy of one salivary gland.

Biology Open • Supplementary information

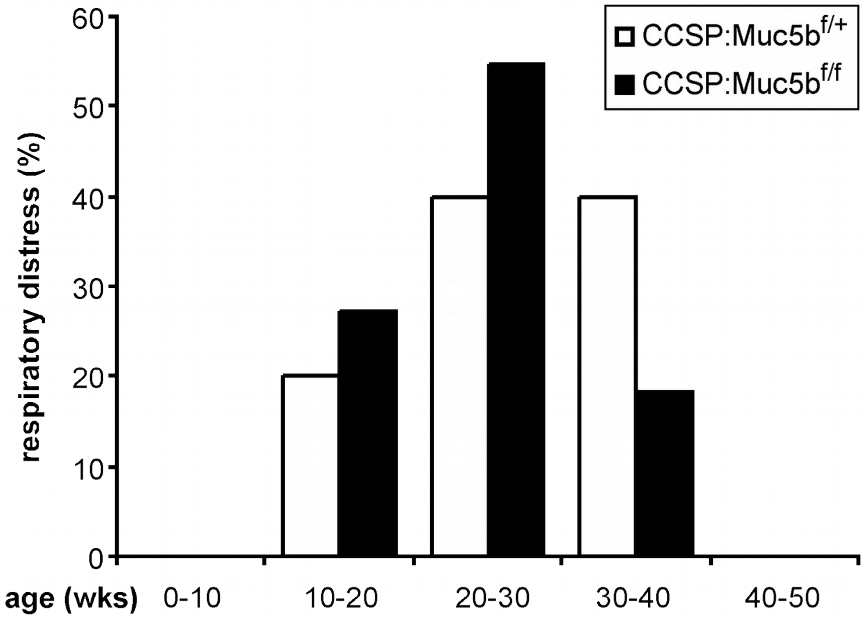

Figure S6. Frequency (%) of CCSPCre-positive mice with respiratory distress and carrying either one or two Muc5b-floxed allele.

Biology Open • Supplementary information

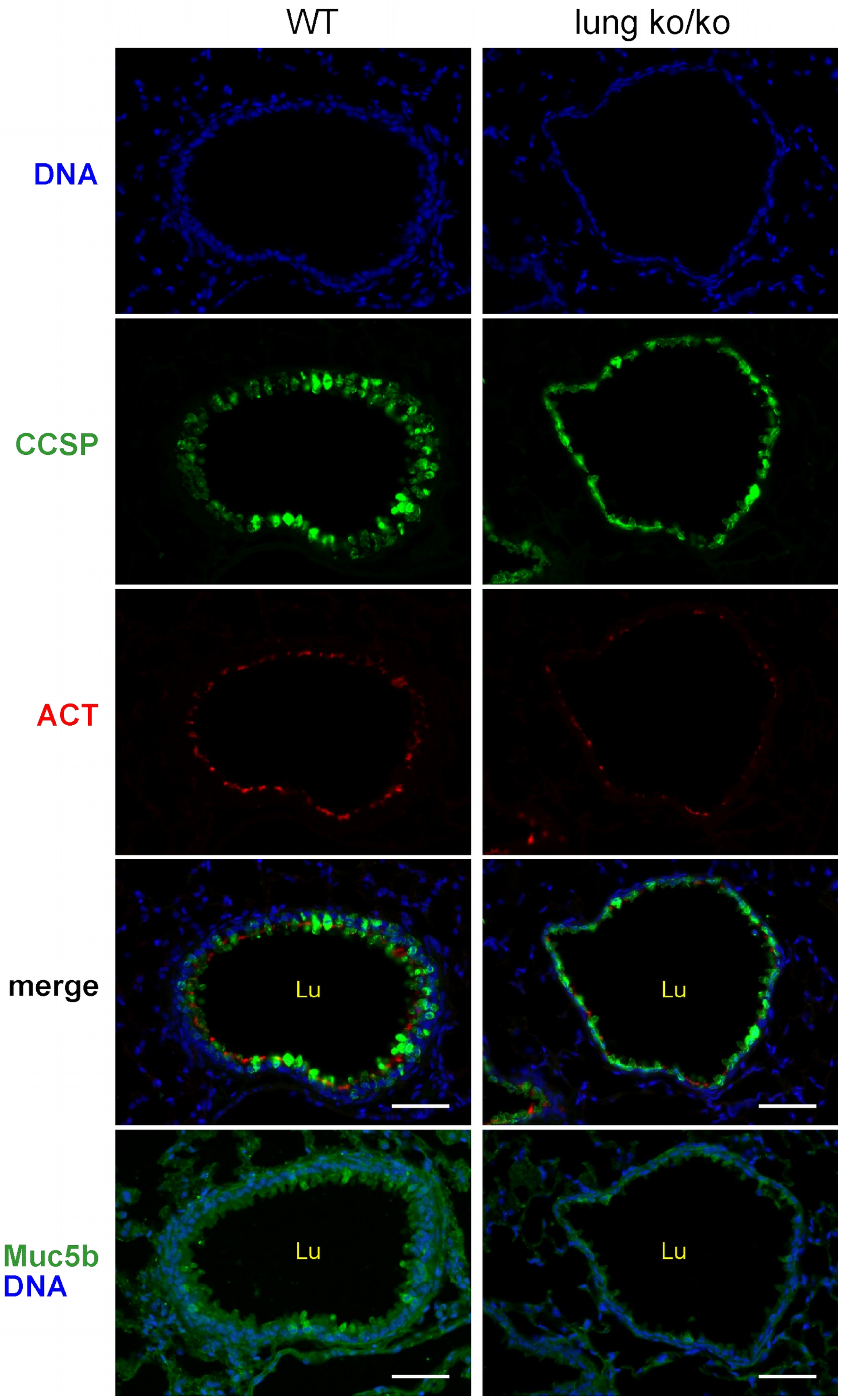

Figure S7. Immunohistochemical analysis of Club cells and ciliated cells. Serial bronchial sections of wild-type (WT) and Muc5b$^{lung\ ko}$ mice were analyzed by immunohistochemical analysis for the detection of CCSP-, ACT- and Muc5b-positive cells. Lu = lumen; Scale bars = 50 µm.

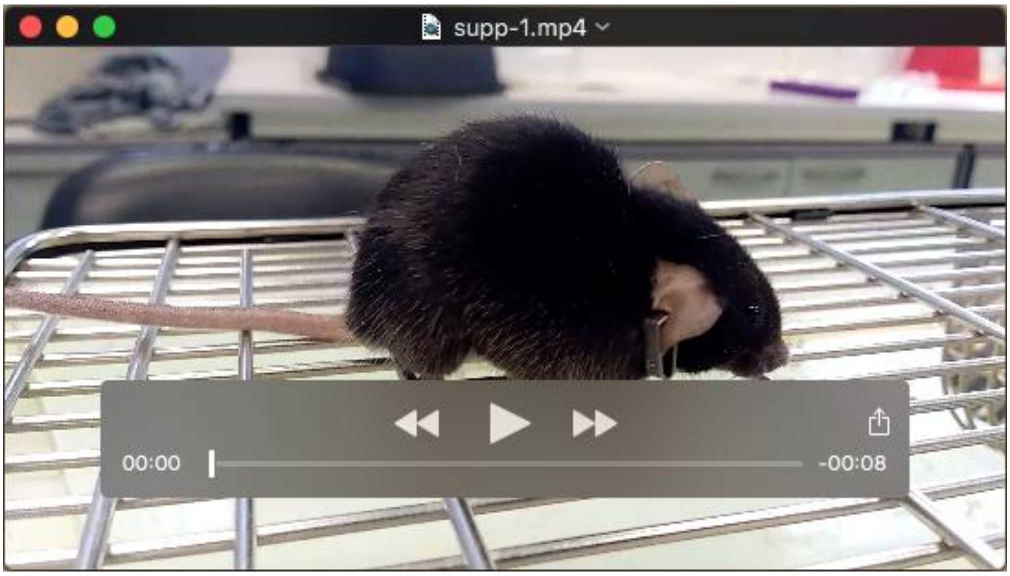

Movie 1. Adult Muc5b$^{ko/+}$ mouse with respiratory distress showing hunched posture, reduced locomotor activity and polypnoea.

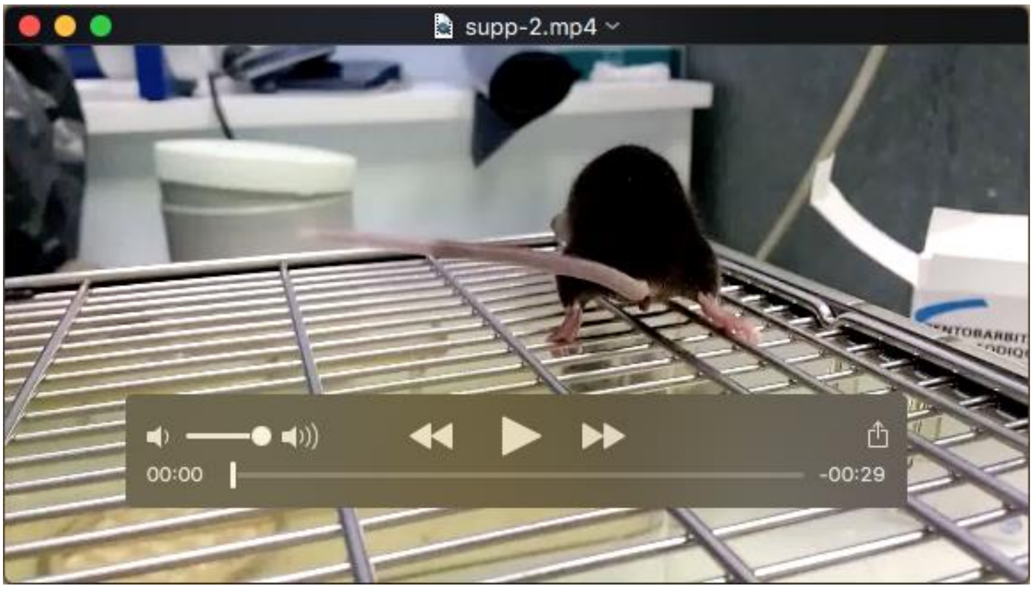

Movie 2. Adult Muc5b$^{ko/+}$ mouse (different from video 1) with respiratory distress showing hunched posture, squeaking and discreet cough.
